# Multi-modal, Multi-measure, and Multi-class Discrimination of ADHD with Hierarchical Feature Extraction and Extreme Learning Machine Using Structural and Functional Brain MRI

**DOI:** 10.3389/fnhum.2017.00157

**Published:** 2017-04-04

**Authors:** Muhammad Naveed Iqbal Qureshi, Jooyoung Oh, Beomjun Min, Hang Joon Jo, Boreom Lee

**Affiliations:** ^1^Department of Biomedical Science and Engineering, Institute of Integrated Technology, Gwangju Institute of Science and TechnologyGwangju, South Korea; ^2^Department of Neuropsychiatry, Seoul National University HospitalSeoul, South Korea; ^3^Department of Neurologic Surgery, Mayo ClinicRochester, MN, USA

**Keywords:** ADHD-200, global functional connectivity, neuroimaging, ANOVA, machine learning, revised recursive feature elimination, hierarchical feature extraction, extreme learning machine

## Abstract

Structural and functional MRI unveil many hidden properties of the human brain. We performed this multi-class classification study on selected subjects from the publically available attention deficit hyperactivity disorder ADHD-200 dataset of patients and healthy children. The dataset has three groups, namely, ADHD inattentive, ADHD combined, and typically developing. We calculated the global averaged functional connectivity maps across the whole cortex to extract anatomical atlas parcellation based features from the resting-state fMRI (rs-fMRI) data and cortical parcellation based features from the structural MRI (sMRI) data. In addition, the preprocessed image volumes from both of these modalities followed an ANOVA analysis separately using all the voxels. This study utilized the average measure from the most significant regions acquired from ANOVA as features for classification in addition to the multi-modal and multi-measure features of structural and functional MRI data. We extracted most discriminative features by hierarchical sparse feature elimination and selection algorithm. These features include cortical thickness, image intensity, volume, cortical thickness standard deviation, surface area, and ANOVA based features respectively. An extreme learning machine performed both the binary and multi-class classifications in comparison with support vector machines. This article reports prediction accuracy of both unimodal and multi-modal features from test data. We achieved 76.190% (*p* < 0.0001) classification accuracy in multi-class settings as well as 92.857% (*p* < 0.0001) classification accuracy in binary settings. In addition, we found ANOVA-based significant regions of the brain that also play a vital role in the classification of ADHD. Thus, from a clinical perspective, this multi-modal group analysis approach with multi-measure features may improve the accuracy of the ADHD differential diagnosis.

## Introduction

The neurodevelopmental disease of attention deficit hyperactivity disorder (ADHD) is among the major health problems both in developing and developed countries of the world. There are no standard biological tests available to diagnose ADHD except behavioral symptoms investigated with psychiatric measures (Arbabshirani et al., 2017). The high (3.4%) prevalence of ADHD among children and adolescents (Polanczyk et al., 2015) makes the automated diagnosis very important. The burden of ADHD on patients, their family, and the societies where they belong is very significant. Typically, the disorder begins to affect the patients from an early age to the entire life span, and without appropriate treatments, the illness leads to poor prognosis. Thus, the early and precise diagnosis of ADHD is very important. Children affected by this disorder have characteristic symptoms such as attention deficit, hyperactivity, and impulsiveness. Currently, it is believed that this characteristic manifestation of symptoms originates from the dysfunction of related cognitive processes (Diamond, 2005). In addition, the underlying mechanisms of ADHD seem to be associated with delayed cortical development (Shaw et al., 2007). The root cause of ADHD is still unknown (Arbabshirani et al., 2017). On the other hand, according to the Diagnostic and Statistical Manual of Mental Disorders, Fifth edition (DSM-5), there are three ADHD subtypes, based on the predominant symptoms: (1) predominantly inattentive presentation, (2) predominantly hyperactive-impulsive presentation, and (3) combined presentation (Association AP, 2013). Over recent years, a rapidly growing number of studies have been published that aim at complementing and improving clinical decision making on the basis of biomarkers derived from different types of data, such as magnetic resonance imaging (MRI) and genomic sequencing. Pattern recognition techniques have shown promising results to detect biomarkers from neuroimaging data. These techniques hold the potential to combine complementary information across different sources in an efficient way (Wolfers et al., 2015). In addition, many previous machine learning based studies investigated these subtypes of ADHD (Solanto, 2000; Castellanos and Tannock, 2002; Colby et al., 2012; Dai et al., 2012; Fair et al., 2012; Igual et al., 2012; Willcutt et al., 2012; Lim et al., 2013; Peng et al., 2013; Johnston et al., 2014; Deshpande et al., 2015; Hammer et al., 2015; Iannaccone et al., 2015; Qureshi and Lee, 2016; Qureshi et al., 2016; Xiao et al., 2016). However, the outcomes from a small number of studies that attempted to distinguish the features of these subtypes with neuroimaging methods were inconclusive (Pineda et al., 2002; Miller et al., 2006). In addition, very few studies including (Qureshi et al., 2016) were conducted on ADHD differential diagnosis (Arbabshirani et al., 2017).

These s/fMRI studies investigated the altered brain activation patterns in ADHD patients and healthy controls. However, the findings across different studies were inconsistent, and the different neural mechanisms between adults and children with ADHD remain unclear (Lei et al., 2015). In fact, the clinical experience of the psychiatrist, a detailed history taking of the patient, and other information resulting from interviews remain important. Pattern recognition techniques deployed in MRI-based neuroimaging studies dates back approximately a decade with the goal of classifying and thereby separating psychiatric patients from controls. However, despite many subsequent efforts, those promising results are not ready for clinical trials beyond research settings yet (Wolfers et al., 2015). However, in the past decade, not only in the psychiatry but also in other medical fields, interest in machine learning increased rapidly. There have been many recent studies using neuroimaging methods in the psychiatric research field (Klöppel et al., 2012; Castro et al., 2014; Schnack et al., 2014). Consequently, with the familiarity and understanding of neuroimaging methods, a pool of candidate features such as cortical thickness, volumetric data, functional connectivity measures, white matter volume, gray matter density, demographic information, and other fMRI data-based features was utilized for machine learning-based classification. Those studies serve as a bridge for the integration of machine learning and neuroscience. The comparatively higher resolution (usually one cubic millimeter or less) of structural MRI data (Arbabshirani et al., 2017) makes it a better choice for the use in classification experiments either as a standalone or as a co-modality in multi-modal experimental settings. The most abundantly used and popular machine learning tool among the neuroimaging community is support vector machine (SVM) (Tenev et al., 2014; Arbabshirani et al., 2017). In this comparative study, we compared an extreme learning machine (ELM) based classification framework with SVMs.

In this study, we focused on the machine learning-based differential diagnosis of the subtypes of ADHD. We utilized an ELM classifier to distinguish ADHD-combined-type (ADHDC) and ADHD-inattentive-type (ADHDI) patients from normally/typically developing children (TDC). In addition, to achieve better classification accuracy, we proposed a modified recursive feature elimination RFE algorithm (Qureshi and Lee, 2016). This algorithm treats cortical and sub-cortical measurement data as an input. The cortical measures include mean cortical thickness, standard deviation of the cortical thickness, surface area, curvature and volume of predefined segments, or anatomical regions (Wee et al., 2013, 2014; Gori et al., 2015). In addition, we also used the sub-cortical measures of structural MRI data including image intensity, white matter volume, and mean volume of the sub-cortical regions. These subcortical measures were not used in our previous study (Qureshi et al., 2016). Global functional connectivity measures from atlas-based cortical regions served as features of the functional MRI data. Moreover, we performed the ANOVA test on both the structural and the functional MRI data and the corresponding significant regional measures served as ANOVA-based features for classification. These ANOVA-based features were acquired in a different manner from the atlas ROI-based features, therefore, we were interested to observe their effect on the classification accuracy. A fully cross-validated, ROI-based data-driven analysis was used for classification.

Several previous studies identified certain numbers of anatomical regions to be affected in ADHD. However, there were very few studies (Arbabshirani et al., 2017) that investigated the broadly different anatomical regions that could play a vital role in the classification of ADHD sub-groups.

## Materials and methods

### Dataset

We obtained neuroimaging data of ADHD patients from the ADHD-200 MRI dataset (Biswal et al., 2010), which is publicly available at the 1,000 Functional Connectomes Project website http://fcon_1000.projects.nitrc.org/indi/adhd200. These T1-weighted structural and resting state functional MRI scans were acquired at six different institutes. The ages of the participants ranged from 7 to 14 years. The sites were Brown University (BU), New York University Child Study Center (NYU), Beijing Normal University (BNU), Kennedy Krieger Institute (KKI), Oregon Health, and Science University (OHSU), and Washington University in St. Louis (WU). All participants have been scanned under 3.0-Tesla scanners. The other technical details about the scanner parameters from each participating site are available at the above-mentioned URL of the ADHD-200 global competition. In addition, all the sites contributing to ADHD-200 had been approved by their local institutional review board (IRB) and complied with local IRB protocols (Qureshi et al., 2016).

#### Subjects

There were approximately 1,000 subjects in the ADHD-200 dataset. However, we followed a balanced design approach and chose 53 subjects exactly as described in (Qureshi et al., 2016) for training from each of the three groups: typically developing children (TDC), ADHD inattentive type (ADHDI), and ADHD combined type (ADHDC). All the training subjects were selected from the training part of the ADHD-200 dataset that consists of 776 subjects. Each group contained nine female and 44 male subjects. There may have existed some intrinsic bias in the data regarding batch effects as well as hardware bias due to multi-site data collection. However, we retained it as default for the criteria of subjects selection for this study similarly to (Qureshi et al., 2016). More details regarding the demographic features of the dataset can be acquired from the same resources listed above. Testing data were separately gathered with matched gender, age, and IQ information from ADHD-200 testing dataset, which corresponded to a pool of 197 subjects. Table 1 summarizes the demographic characteristics of our selected training and testing subjects.

**Table 1:** **Demographic variables of the participant subjects for training and testing**.

**Groups**	**TDC**	**ADHDI**	**ADHDC**
**TRAINING**			
No. of subjects	53	53	53
Age (mean ± SD)	12.75 ± 3.86	12.42 ± 2.23	11.83 ± 3.52
Full IQ (mean ± SD)	114.86 ± 13.86	102.47 ± 13.11	110.10 ± 13.88
Handedness	Right only	Right only	Right only
**TESTING**			
No. of subjects	14	14	14
Age (mean ± SD)	11.35 ± 1.69	11.75 ± 1.97	10.30± 1.56
Full IQ (mean ± SD)	118.86 ± 6.34	108.29 ± 7.85	115.20 ± 13.64
Handedness	Right only	Right only	Right only

*TDC, typically developing children; ADHDI, attention-deficit/hyperactivity disorder, inattentive type; ADHDC, attention-deficit/hyperactivity disorder, combined type; SD, standard deviation*.

### Preprocessing of structural MRI data

Cortical reconstruction and volumetric segmentation were performed with the FreeSurfer v5.3.0 image analysis suite, which is documented and freely available for download online at http://surfer.nmr.mgh.harvard.edu/. The technical details of these procedures have been described elsewhere (Dale and Sereno, 1993; Dale et al., 1999; Fischl et al., 1999a,b, 2001, 2002, 2004a,b; Fischl and Dale, 2000; Ségonne et al., 2004; Han et al., 2006; Jovicich et al., 2006; Reuter et al., 2010, 2012). Briefly, the preprocessing procedure includes motion correction of volumetric T1-weighted images, removal of non-brain tissue using a hybrid watershed/surface deformation procedure (Ségonne et al., 2004), and automated Talairach transformation. Moreover, the procedure includes the segmentation of the subcortical white matter and deep gray matter volumetric structures including hippocampus, amygdala, caudate, putamen, ventricles (Fischl et al., 2002, 2004a), intensity normalization (Sled et al., 1998), tessellation of the gray matter-white matter boundary, and automated topology correction (Fischl et al., 2001; Ségonne et al., 2007). In addition, surface deformation following intensity gradients was performed to optimally place the gray/white and gray/cerebrospinal fluid borders at the location where the greatest shift in intensity defines the transition to the other tissue class (Dale and Sereno, 1993; Dale et al., 1999; Fischl and Dale, 2000). Once the cortical models are complete, a number of deformable procedures can be performed for further data processing and analysis. These procedures include surface inflation (Fischl et al., 1999a), registration to a spherical atlas that is based on individual cortical folding patterns to match the cortical geometry across subjects (Fischl et al., 1999b). Moreover, parcellation of the cerebral cortex into units with respect to gyral and sulcal structure (Fischl et al., 2004b; Desikan et al., 2006) and creation of a variety of surface based data including maps of curvature and sulcal depth are part of the procedure. This method uses both intensity and continuity information from the entire three-dimensional MR volume in segmentation and deformation procedures to produce representations of cortical thickness, calculated as the closest distance from the gray/white boundary to the gray/CSF boundary at each vertex on the tessellated surface (Fischl and Dale, 2000). The maps are created using spatial intensity gradients across tissue classes rather than simply relying on absolute signal intensity. The maps produced are not restricted to the voxel resolution of the original data, and are thus capable of detecting submillimeter differences between groups. Procedures for the measurement of cortical thickness have been validated against histological analysis (Rosas et al., 2002) and manual measurements (Kuperberg et al., 2003; Salat et al., 2004). FreeSurfer morphometric procedures have been demonstrated to show good test-retest reliability across scanner manufacturers and across field strengths (Han et al., 2006; Reuter et al., 2012). A cortical surface-based Desikan-Killiany-Tourville (DKT) atlas Klein and Tourville, 2012) was mapped to a sphere aligning the cortical folding patterns, which provided accurate matching of the morphologically homologous cortical locations across subjects. For each of the DKT31 protocol-based segments, FreeSurfer calculated the nine different measures, including number of vertices, surface area, gray matter volume, average cortical thickness, cortical thickness standard deviation, cortical mean curvature, cortical Gaussian curvature, cortical folding index, and cortical curvature indices (Colby et al., 2012). For the subcortical regions, FreeSurfer calculated the area and volume of the whole segment, white matter volume, intensity and overall volume of the whole brain divisions including cerebrospinal fluid (CSF), intracranial volume (ICV), gray matter (GM), and white matter (WM). Two of the selected measures are the most common features in the structural studies (Arbabshirani et al., 2017). The surface area was calculated by computing the area of every triangle in a standardized spherical surface tessellation. The local curvature was computed using the registration surface based on the folding patterns (Qureshi et al., 2016).

#### Cortical and subcortical features

We used nine of the above-mentioned measures including cortical thickness, surface area, mean curvature, volume, and cortical thickness standard deviation. Measures used as the structural ROI features included whole segment volume, white matter volume and intensity from subcortical regions, and overall brain volumes. In addition, After the preprocessing, FreeSurfer's QA Tools were used for the detection and removal of the outliers and negative features.

### Preprocessing of resting state functional MRI data

Preprocessing of functional MRI data was based on Analysis of Functional Neuroimages AFNI software; http://afni.nimh.nih.gov/afni/ (Cox, 1996). Every single echo planar image (EPI) volume was co-registered to the corresponding anatomical image of the subject and mapped to Talairach coordinates space with the TT_N27+tlrc template. We excluded the first six images from each EPI volume to achieve the MR steady state. In addition, slice-timing correction was performed. We censored and cut out time points based on their number of outliers and head motion magnitude. Same number of slices were cut out for all subjects. Slice alignment was applied by using the local Pearson's correlation (LPC) cost function. The correction of head motion along with averaging the EPI volumes was performed to obtain a mean functional image. The dataset was already reoriented to right posterior inferior (RPI) in the ADHD-200 dataset repository. Each EPI volume underwent the linear multiple regression to regress the motion derivatives and effects of the white matter and cerebrospinal fluids. Spatial smoothing was performed by using a Gaussian kernel with a blur size of 6-mm full width at half maximum (FWHM). A polynomial detrending was applied. AFNI program 3dmaskave was used to calculate the beta values for the correlation analysis. A run of 10,000 Monte Carlo simulations was conducted with AlphaSim program. The cluster size of 10 voxels was determined at a family-wise error rate corrected with *p* < 0.000001 to avoid the problem of multiple comparisons. As a result, the clusters consisting of lower than 10 voxels were discarded from the analysis.

#### Global connectivity features

**Figure 1:**
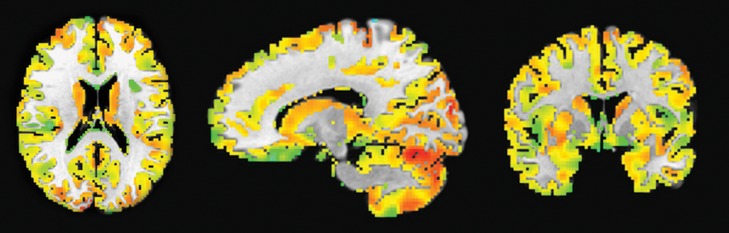
**A typical global functional connectivity map for a single ADHD patient**. The left column shows the axial view; middle column shows the sagittal while the rightmost column shows the coronal view. The color temperature in the connectivity maps represents the strength of the connectivity measure between different resting-state networks in the cortical region.

Global connectivity measure was used to calculate the average brain-wise correlation coefficients (GCOR) of all the possible combinations of voxel time series. The GCOR estimation of the cortical regions is a computationally expensive process. It involves the calculation of M (M-1)/2 correlation estimates for an M voxels volume (Saad et al., 2013). AFNI simplifies this problem by taking de-meaned time series of each voxel and scaling it by its Euclidean norm. In addition, it averages the scaled time series over the whole brain mask and finally the length (*l*_2_ -norm) of this averaged series represents the GCOR. ADHD is considered as an age-related neurodevelopmental disorder and the symptoms might reflect age-related differences in the cortical and subcortical maturation that characterize ADHD (Xiao et al., 2016). Therefore, we first made a gray matter mask of 5-mm thickness created by using AFNI program 3dSeg and 3dcalc to acquire the functional connectivity maps of only the cortical regions of the brain. The clean EPI data were resampled with the gray matter mask to obtain the data only within the cortical regions. We used AFNI program 3dTcorrMap to get the global functional connectivity maps. This involved the calculation of correlation (Pearson's r) of the residual time series in each voxel with every other voxel in their brain mask and recording the mean correlation back in the voxel. These connectedness values were further transformed with Fisher's z-transformation to yield normally distributed measures (Gotts et al., 2012). We used DKD_ Desai_PM Atlas (Desikan et al., 2006) of AFNI package to acquire the ROI measures from the 102 cortical regions across the whole brain. Figure 1 depicts a typical global functional connectivity map for a single ADHD patient. A summary of all the atlas-based structural and functional ROI features is presented in Table 2.

Table 2**Summary of the structural and functional atlas-based ROI, and ANOVA ROI features**.

**Table:** 

**Structural**		**Functional**		
**DKT Atlas-based ROI Measures**	**Count**	**DKD_Desai_PM Atlas- based ROI Measures**		**Count**
Cortical thickness	64	Average global connectivity		102
Cortical thickness SD	62			
Surface area	64			
Volume	62			
Curvature	62			
White matter volume	68			
Sub-cortical volume	37			
Sub-cortical intensity	40			
Whole brain volume	16			
Total unimodal features	475			102

**Table:** 

**Multi-measure features**			
**Modality**	**Structural**	**Functional**
Atlas ROI-based	475	102
ANOVA ROI-based	12	9
Total unimodal Features	487	111
Total Multi-modal Features	598	

*ROI, Region of interest; ANOVA, analysis of variance; SD, standard deviation. The atlas-based ROI feature count for structural data was obtained by following the same procedure as used in Qureshi et al. (2016). The functional data feature count was based on the number of ROI obtained by DKD_Desai_PM atlas (Desikan et al., 2006) from AFNI software package. Table S1. (Supporting Information) describes the features name and count in detail*.

### Statistical analysis

After preprocessing, the structural data were converted into surface maps for ANOVA analysis. Heat kernel smoothing was applied on the dataset after conversion. An AFNI program 3dANOVA2 (Cox, 1996) was used for this analysis.

First, to examine the differences among the three groups, the multiple comparisons test was performed using the AFNI program 3drefit with the FDR correction on both the structural and the functional data. In addition, for the structural data, the AFNI program SurfClust was used to find regions that have significant differences between groups with an area of more than 50 mm^2^. The *p*-value threshold was set to be 0.001 (Qureshi et al., 2016). This was followed by a *post-hoc t*-test to evaluate the actual differences among the three groups. For the functional data, we used AFNI program 3dclust to generate the mask of significant ROIs of ANOVA analysis. In addition, 3dROIstats was used with this mask to acquire the average connectivity value from each subject. Since these data were very significant, we set the *p*-value at a very low level of 0.000001. Finally, we extracted 12 significant regions from the structural data and nine significant regions from the functional data that served as the ANOVA-based features for classification. It is worth noting that the ANOVA analysis was performed only on the training dataset to avoid the potential bias (Arbabshirani et al., 2017) of the overall accuracy of the classifier.

#### ANOVA ROI based features

The mean measures of the significant regions acquired from the ANOVA analysis was used as the features for the classification. Table 2 describes briefly the ANOVA ROI based features count.

### Pearson correlation coefficient of ANOVA-based significant features

The Pearson correlation coefficients were calculated between structural and functional ANOVA-based significant ROIs and age separately. For the structural data, we extracted the average cortical thickness of each ROI for each individual subject using the AFNI program 3dmaskave. The *p*-value of the correlation coefficients was calculated based on our sample size (Rosenhead, 1963). For the functional data, we calculated the average beta value of each ANOVA-based ROI of each individual subject using AFNI program 3dmaskave.

### Hierarchical feature selection and ranking

In neuroimaging, pattern recognition is sometimes referred to as multi-voxel pattern analysis (MVPA), as voxels are often used directly as features. The features can be categorized as voxel, region, or network-derived set of values. In a voxel-based feature set, features are extracted on the voxel level. In a region-based feature set, features are derived by parcellation of brain images from predefined regions based on anatomical or functional brain atlas. In a network-based feature set, features are extracted by combining voxels across networks, like the functional connectivity based features (Wolfers et al., 2015). We used the last two approaches (region and network driven features) in this study in addition to an uncommon approach of statistical measurement (ANOVA) based feature extraction. The region-based feature selection is the most common approach used in the fMRI studies (Arbabshirani et al., 2017).

In this study, the multi-measure features were acquired from the significant regions obtained by ANOVA analysis and atlas-based ROIs separately for both structural and functional MRI datasets. Finally, we gathered a big pool of features as shown in Table 2. Therefore, optimal feature selection was necessary to get a high classification accuracy.

#### Feature optimization

Optimal feature selection provides a subset of features that leads toward the optimal classification accuracy (Qureshi et al., 2016). We used a hierarchical approach to optimize the features for the best classification accuracy. Both the filter- and wrapper-based feature selection models were used in this study. Specifically, this study utilizes a combination of two feature selection methods, namely the revised recursive feature elimination (rRFE) proposed by Qureshi and Lee (2016) and the least absolute shrinkage and selection operator (LASSO).

#### LASSO

A feature selection method that utilizes the objective optimization can be conveniently formulated as convex optimization problems with global optimal solutions. A good example of an objective function with an error term and a regularization term is the least absolute shrinkage and selection operator (LASSO) based regularized regression for features selection. We used Matlab implementation of lasso regularization with the regularization coefficient λ in this study. The value of λ was optimized by grid search method. For a given value of λ which is a non-negative parameter, lasso solves the problem:

$$min_{\gamma_{0},\;\gamma }\left(\frac{1}{2n}\;\underset{i=1}{\sum^{n}}\;{\left(u_{i}-\;\gamma_{o}-\;x_{i}^{T}\gamma \right)}^{2}+\;\lambda \underset{j=1}{\sum^{q}}\;\left|\gamma_{j}\right|\right)$$

Where *n* is the number of observations. *u*_*i*_ is the response at observation *i*. ***x***_***i***_ is input data, a vector of *q* values at observation *i*. λ is a non-negative regularization parameter. The parameters γ_*o*_ and **γ** are scalar and *q*-vector respectively. As λ increases, the number of nonzero components of **γ** decreases. The lasso problem involves the *l*_1_ norm of **γ** (Tibshirani, 1996; Hastie et al., 2009; Friedman et al., 2010). After selection based on the cross-validated LASSO, selected features were finally fed to the rRFE algorithm for final ranking.

#### Revised recursive feature elimination (rRFE)

Recursive features elimination based on linear SVM (RFE-SVM) is a well-known wrapper model for feature optimization. A wrapper model usually consists of two steps, (1) feature subset selection using the accuracy of the base classifier, and (2) learning and testing with the best feature subset. The wrapper approach utilizes the prediction performance of a base classifier. It performs selection by using the classifier as a black box and rank the subset of features by their predictive power (Yusta, 2009). The preselected features further fed into a wrapper model rRFE to generate the feature-ranking list according to the order of significance. In this study, we used an rRFE algorithm (Qureshi and Lee, 2016), a recently proposed modified version of the original RFE-SVM. A more detailed justification for choosing this method for feature selection can be found in Qureshi et al. (2016).

### Classification

Two classifiers were used in this study, namely, linear extreme learning machine (ELM) and support vector machine (SVM) with linear and radial basis function (RBF) kernels. The classifiers were used in both binary and multi-class settings. In the case of multi-class, we used the one versus all (OVA) approach. In addition, we also mentioned the ELM-based classification results without applying the feature selection to validate the significance of the proposed feature selection framework. A brief description of both classifiers used in this study is as following.

#### Extreme learning machine classifier

Extreme learning machine originally proposed by Huang et al. (2006) has been adopted in many previous neuroimaging studies (Termenon et al., 2013; Zhang et al., 2015) in the binary and multi-class settings (Huang et al., 2012). However, to the best of our knowledge, the present study is the first in this domain that utilized sigmoid activation function based ELM for the multi-class, multi-measure, and multi-modal classification of neuroimaging data. ELM randomly assigns the weights and bias to the input data to compute the output weight matrix. This random assignment of weights makes the ELM algorithm very fast as compared to SVMs. A more detailed discussion of the classifier can be found elsewhere (Huang et al., 2006; Qureshi et al., 2016). The ELM classifier requires the “number of nodes” as the only hyper-parameter that needs to be tuned for achieving the maximum performance in terms of accuracy. In this study, we used the Matlab implementation of the ELM. A greedy search method was used to tune this parameter for achieving the maximum test accuracy. In this study, the search scale for selecting this parameter was set to *N* = [1, 2, …, 200]. All the features were normalized and scaled to the values between -1 and 1 for the better performance of ELM classifier (Qureshi et al., 2016).

#### SVM classifier

SVM, which was originally proposed by Cortes and Vapnik (1995), considered as one of the most popular machine-learning tools in the neuroscience domain in the last decade. It is a supervised classification algorithm. We used this algorithm in both binary and multi-class (more commonly known as one-versus-all or OVA) settings. It maps features in higher dimensional space using linear and nonlinear functions known as kernels. In this study we used both linear and nonlinear radial basis function (RBF) kernels (Qureshi et al., 2016). The only parameter that needs to be tuned in SVM is the value of regularization parameter “σ” while utilizing the radial basis function (RBF) kernel. We used a greedy search method to tune this parameter for achieving the maximum test accuracy. In this study, the search scale for selecting this parameter was set to σ = [0.1, 0.2, …, 2.0].

### Performance evaluation and significance testing methods

The classifier performance was measured in terms of classification accuracy and Kappa score for multi-class classification. Classification accuracy was determined as

$$\begin{array}{l} Accuracy=\;\frac{\left(TP+TN\right)}{\left(TP+FP+TN+FN\right)}*100, \end{array}$$

Where TP is true positive, TN is true negative, FP is false positive and FN is false negative. These values were obtained by computing the confusion matrix (Qureshi et al., 2016). Kappa score is considered as a robust statistical measure for the assessment of multi-class classifiers. It is defined by Cohen (1960) as

$$\begin{array}{l} k\;=\;Pr(a)-Pr(e)\;/\;1-Pr(e), \end{array}$$

Where *Pr*(*a*) is the relative observer agreement between raters and *Pr*(*e*) is hypothetical probability of chance agreement. The value of *k* ranges between +1 and −1, where *k* = 1 represents perfect classification, *k* = 0 represents chance level, and *k* = −1 represents completely erroneous classification (Wang et al., 2012; Qureshi et al., 2016).

A Permutation test was used to assess the statistical significance of the ELM classifier performance (Golland and Fischl, 2003). Briefly, it works as follows. First, we choose the actual test accuracy as the test statistic of the classifier, the class labels for testing dataset permuted randomly and given to the classifier and check for the cross validation. Generally, the lower *p*-value of the permuted prediction rate against the prediction rate with the original data labels indicates the higher significance of the classifier performance. There is no fixed rule for setting the number of permutations. We have permuted the data 10,000 times in the current study (Qureshi et al., 2016).

**Figure 2:**
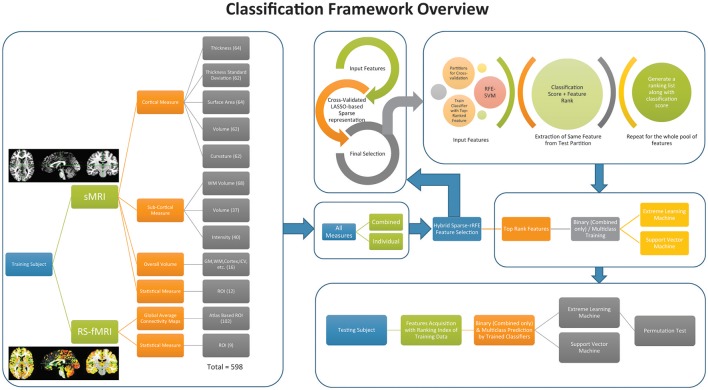
**Overall classification framework of the current study**. The box at the extreme left presents the feature acquisition. Right, top four boxes represent the feature selection and training of the classifier. The box at the bottom on the extreme right shows the framework to acquire the testing accuracy from the data.

Figure 2 depicts the overview of the classification framework of this study.

## Results

We used ANOVA analysis to extract the features for classification in this study along with the ROI based features as proposed in a recent study (Qureshi et al., 2016). These ANOVA-based features were gathered separately from both structural and functional MRI datasets. Classification accuracy was measured for both unimodal and multi-modal features. The following explanation of the ANOVA results of each modality describes those ANOVA-based features briefly.

### Structural ANOVA

**Figure 3:**
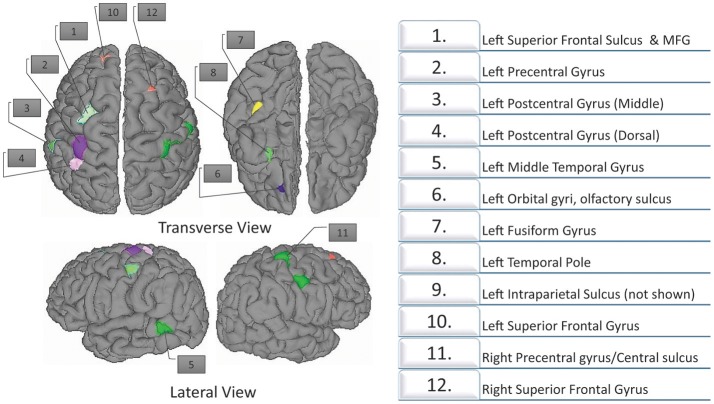
**Twelve regions with significant differences as determined by structural ANOVA**. The top row shows the transverse views. The bottom figures show the lateral views. The most significant results are located in the superior frontal gyrus.

ANOVA analysis was applied to the cortical thickness measures acquired by FreeSurfer and further smoothed with Heat kernel by AFNI preprocessing pipelines. We used only cortical thickness measures for the ANOVA analysis as it is the most important structural imaging biomarker (Shaw et al., 2007; Narr et al., 2009). A basic description of this results was presented briefly in a recent previous work (Qureshi et al., 2016). In the present study, we are using the ANOVA results for extracting the features for classification. The ANOVA tests showed (see Figure 3) 10 areas of significant thickness change in the left hemisphere and two areas in the right hemisphere (corrected at *p* < 0.001, cluster size 50 mm^2^). AlphaSim was applied on the result for correction. Table 3 summarizes our findings.

**Table 3:** **Twelve regions with significant differences, based on ANOVA (corrected at ***p*** < 0.001, cluster size 50mm^**2**^)**.

	**Cluster size (mm^2^)**	**Coordinates**			**Average Cortical Thickness**		
		***x***	***y***	***z***	**ADHDI**	**ADHDC**	**TDC**
**LEFT HEMISPHERE**							
Superior frontal sulcus, MFG	263.40	−24.38	10.534	57.315	2.6962	2.5483	2.7053^*^
Precentral gyrus	226.75	−28.876	−24.375	59.984	2.6088	2.5351	2.7823^*^
Postcentral gyrus (middle)	120.55	−51.721	−20.515	48.220	2.0511	2.0943	2.2158^*^
Postcentral gyrus (dorsal)	108.48	−31.086	−33.537	61.708	1.9221	2.0749	2.2061^*^
MTG	97.22	−56.836	−43.266	−0.973	3.0002	3.0167	3.1930^*^
Orbital gyri, olfactory sulcus	92.90	−16.190	43.782	−6.448	2.3751^*^	2.2694	2.1542
fusiform gyrus	85.49	−34.077	−24.091	−16.101	3.0491^*^	2.8391	2.9911
Temporal pole	72.70	−25.916	15.919	−22.011	3.7693	3.4891	3.8485^*^
Intraparietal sulcus	70.85	−18.823	−55.081	35.649	2.1532	2.2230	2.3272^*^
SFG	61.23	−16.234	52.671	40.944	3.0485^*^	2.9041	2.8155
**RIGHT HEMISPHERE**							
Precentral gyrus/Central sulcus	686.22	38.521	−14.525	50.407	2.5374	2.5437	2.6961^*^
SFG	69.90	14.811	26.247	58.640	3.0023^*^	2.8072	2.9575

*TDC, typically developing children; ADHDI, attention-deficit/hyperactivity disorder, inattentive type; ADHDC, attention deficit/hyperactivity disorder, combined type; MFG, middle frontal gyrus; MTG, middle temporal gyrus; SFG, superior frontal gyrus*.

* *Indicates the highest cortical thickness value of the region among the three groups*.

### Functional ANOVA

**Figure 4:**
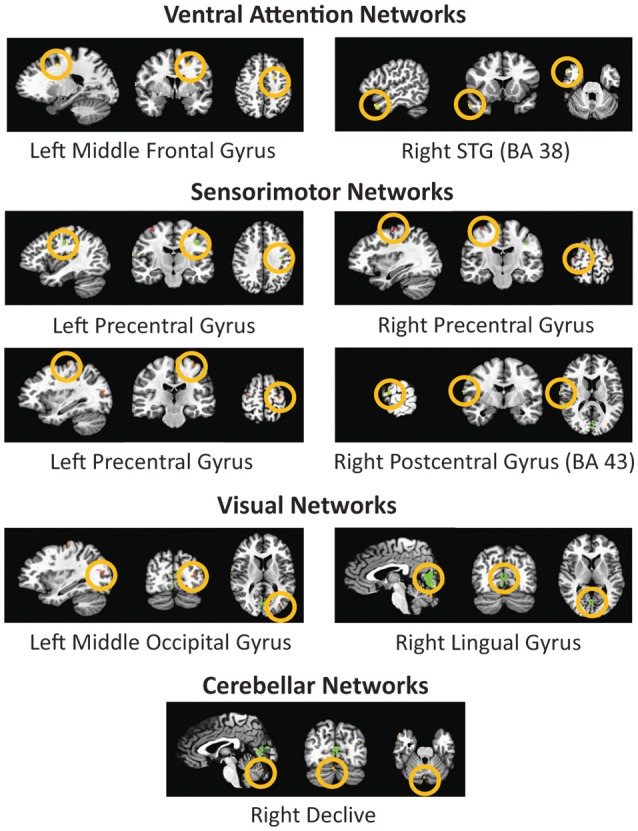
**Four significant networks including nine significant sub-network regions acquired by ANOVA analysis of the global connectivity maps of resting state fMRI data**. The figure depicts the selected region in all three sagittal, coronal, and axial views respectively.

The ANOVA tests of resting state fMRI data showed nine areas of significant change (corrected at *p* < 0.000001, cluster size 11 mm^2^) (see Figure 4) from four significant functional networks of the human brain including the ventral attention network, sensorimotor network, visual network, and cerebellar network. Table 4 summarizes the details of the significant regions.

**Table 4:** **Nine regions with significant differences, based on functional ANOVA (corrected at ***P*** < 0.000001, and cluster size 11 mm^**2**^)**.

**Atlas regions**	**Cluster size (mm^2^)**	**coordinates**		
		***x***	***y***	***z***
Right Lingual Gyrus	186	−3.0	71.0	−4.0
Left Precentral Gyrus	30	37.0	15.0	34.0
Right Precentral Gyrus	26	63.0	5.0	12.0
Right STG (BA 38)	23	−51.0	−15.0	−26.0
Left Middle Frontal Gyrus	18	21.0	1.0	42.0
Right Declive	13	−3.0	73.0	−20.0
Left Precentral Gyrus	13	29.0	21.0	58.0
Left Middle Occipital Gyrus	11	31.0	75.0	16.0
Right Postcentral Gyrus (BA 43)	11	−65.0	14.0	15.0

*STG, superior temporal gyrus; BA, Brodmann area*.

Our classification results confirm that these regions can be used as potential biomarkers/features for the multi-class classification of ADHD s/fMRI dataset. The ANOVA-based features gave high classification accuracy both as standalone modality and in combination with ROI-based features.

### Multi-class classification

Our results suggest that the optimized features are robust and yield higher classification accuracies both in binary and multi-class classification settings. We achieved a maximum of 76.190% accuracy (*p* < 0.0001) on the test data by using the proposed hierarchical feature extraction framework with (ELM) in multi-class settings. Table 5 summarizes the overall multi-class (one vs. all settings) accuracies, kappa score, and *p*-values of structural, functional, multi-modal, and multi-measure data.

**Table 5:** **Multi-class classification results**.

**Structural data**												
**Classifier**		**Measure type (feature count)**										
		**CT (64)**	**Area (62)**	**Curvature (62)**	**Volume (62)**	**CTSD (62)**	**SCV (37)**	**WMV (68)**	**SCI (40)**	**OV (16)**	**All ROI (475)**	**ABR (12)**
ELM	Accuracy (%)	69.048	64.286	66.667	69.048	71.429	59.524	66.667	64.286	61.905	**73.81**	66.667
	Kappa score	0.5357	0.4643	0.5	0.5357	0.5714	0.3929	0.5	0.4643	0.4286	**0.6071**	0.5
	*p*-value	<0.0001	<0.0001	<0.0001	<0.0001	<0.0001	<0.0001	<0.0001	<0.0001	<0.0001	<**0.0001**	<0.0001
ELM-NFS	Accuracy (%)	57.143	47.62	52.381	50	50	42.858	52.381	47.62	45.239	50	50
	Kappa score	0.3571	0.2143	0.2857	0.25	0.25	0.1429	0.2858	0.2143	0.1786	0.25	0.25
	*p*-value	<0.0004	<0.0180	<0.0016	<0.0089	<0.0065	<0.0716	<0.0035	<0.0140	<0.0346	<0.0046	<0.0062
SVM linear	Accuracy (%)	54.762	47.62	61.905	50	54.762	45.239	52.381	57.143	50	50	42.858
	Kappa score	0.3214	0.2143	0.4286	0.25	0.3214	0.1786	0.2858	0.3571	0.25	0.25	0.1429
SVM-RBF	Accuracy (%)	52.381	47.62	33.333	47.62	50	42.857	50	47.62	42.858	52.381	40.477
	Kappa score	0.2857	0.2143	0	0.2143	0.25	0.1429	0.25	0.2143	0.1429	0.2857	0.1071
**Functional data**												
**Classifier**						**Measure type (feature count)**						
						**Average global Connectivity (102)**						**ABR (09)**
ELM			Accuracy (%)		**71.429**							64.286
			Kappa score		**0.5714**							0.4643
			*p-value*		<**0.0001**							<0.0001
ELM-NFS			Accuracy (%)		59.524							64.286
			Kappa score		0.3929							0.4643
			*p-value*		<0.0001							<0.0001
SVM linear			Accuracy (%)		61.905							52.381
			Kappa score		0.4286							0.2857
SVM-RBF			Accuracy (%)		42.857							40.476
			Kappa score		0.1429							0.1071
**Multi-modal and Multi-measure Data**												
**Classifier**						**Measure type (feature count)**						
						**ABR (21)**		**All ROI (577)**			**All measures (598)**	
ELM		Accuracy (%)				69.048		71.429			**76.19**	
		Kappa score				0.5357		0.5714			**0.6429**	
		*p-value*				<0.0001		<0.0001			<**0.0001**	
ELM-NFS		Accuracy (%)				52.381		52.381			57.1429	
		Kappa score				0.2857		0.2857			0.3571	
		*p-value*				<0.0035		<0.0021			<0.0001	
SVM Linear		Accuracy (%)				54.762		64.286			64.286	
		Kappa score				0.3214		0.4643			0.4643	
SVM-RBF		Accuracy (%)				52.381		54.762			57.143	
		Kappa score				0.2857		0.3214			0.3571	

*CT, cortical thickness; CTSD, cortical thickness standard deviation; SCV, sub-cortical volume; WMV, white matter volume; SCI, sub-cortical intensity; OV, overall volume; ROI, region of interest; ABR, analysis of variance (ANOVA) based ROI; ELM, extreme learning machine; NFS, no feature selection; RBF, radial basis function. Besides the ELM-NFS all the three (ELM, SVM linear and SVM-RBF) based classification scores were obtained with the most discriminative features selected through the hierarchical feature selection method. Table S1. in (Supporting Information) describes the features name and count in detail. Bold values represents the highest accuracy and its corresponding evaluation measures*.

### Binary classification results

Binary classification results are presented in the current study to demonstrate the fact that, ideally, multi-class classification results show equaling and generally lower accuracy than results from the binary classification of the same dataset (Qureshi et al., 2016).

The major result of the current study is the highest prediction accuracy of the ADHD dataset using all the measures and modalities together as features; therefore, we performed the binary classification on the combined measures only. In binary classification settings, we achieved a maximum of 92.857% (*p* < 0.0001) prediction accuracy. It is interesting to note that the proposed feature selection framework selected only structural features from multi-modal input to acquire this accuracy. Most significant feature measures were mean cortical thickness, volume, area, and standard deviation of cortical thickness respectively. Even though the functional features were not selected in the final selection but they assist the selection algorithm to extract the most discriminative structural features (both ROI and ANOVA-based) from the pool. When we used only the structural features for binary classification we could not reach the same accuracy as we did while using the multi-modal features. In addition, we were not able to achieve this high accuracy in the previous work (Qureshi et al., 2016) because all of the cortical feature measure types utilized in the present study were not used in it. We also learned from the previous study (Qureshi et al., 2016) that many of the feature measures do not have any predictive power including folding and curvature indices, therefore we did not include those measures in this study. Briefly, we achieved the following binary accuracies with each individual modality. With only structural ANOVA features 89.29%; with only functional ANOVA features 82.14%; with only functional ROIs 85.17%, and with only structural ROIs 85.30%.

Table 6 summarizes the classification scores of all the disease groups along-with sensitivity, specificity, F1-score, recall, and precision measures for all the multi-modal and multi-measure features.

**Table 6:** **Binary classification results**.

**Classifier**		**Group**		
		**ADHDC-TDC**	**ADHDC-ADHDI**	**ADHDI-TDC**
ELM	Accuracy (%)	89.286	85.714	**92.857**
	*p*-value	<0.0001	<0.0001	<**0.0001**
	Sensitivity	92.857	100.00	**85.714**
	Specificity	85.714	71.429	**100.00**
	F1-score	89.655	87.500	**92.307**
	Recall	92.857	71.429	**100.00**
	Precision	86.667	100.00	**85.714**
ELM-NFS	Accuracy (%)	71.429	67.857	67.857
	*p*-value	<0.0351	<0.0348	<0.0343
	Sensitivity	100.00	85.714	64.286
	Specificity	64.286	50.000	71.429
	F1-score	78.571	72.727	66.667
	Recall	42.857	85.714	64.286
	Precision	64.286	63.158	69.231
SVM linear	Accuracy (%)	71.429	82.143	67.857
	Sensitivity	64.285	92.857	42.857
	Specificity	78.571	71.428	92.857
	F1-score	69.231	83.839	57.143
	Recall	64.286	92.857	42.857
	Precision	75.000	76.471	85.714
SVM-RBF	Accuracy (%)	53.571	57.143	60.714
	Sensitivity	7.1429	100.00	78.571
	Specificity	100.00	14.285	42.857
	F1-score	0.1333	70.000	66.667
	Recall	100.00	100.00	78.571
	Precision	7.1429	53.846	57.894

*ELM, extreme learning machine; TDC, typically developing children; ADHDI, attention-deficit/hyperactivity disorder-inattentive type; ADHDC, attention-deficit/hyperactivity disorder combined type; SVM, support vector machine; RBF, radial basis function; NFS, no feature selection applied. Besides the ELM-NFS all the three (ELM, SVM linear, and SVM-RBF) based classification scores were obtained with the most discriminative features selected through the hierarchical feature selection method. Bold values represents the highest accuracy and its corresponding evaluation measures*.

It is important to note that two of the three ELM classifier-based binary classification scores (ADHDC-TDC and ADHDI-TDC) without feature selection overlap exactly with the accuracy of the linear SVM with the most discriminative features. In other words, we can say that the lowest accuracy of the ELM classifier overlaps with the highest accuracy of the SVM classifier.

### Correlation coefficient: structural data

After applying the Bonferroni correction method with *p* < 0.000463, we found two regions that were negatively correlated with age in TDC group (Table 7). These regions were left middle temporal gyrus and left superior frontal gyrus. On the other hand, some individual IQ and symptoms severity score data were not included in the original database. Therefore, we only use the subjects with available IQ and symptoms severity score information to perform the calculation of the correlation coefficients. Those subjects with missing IQ information included nine from the TDC group and 13 from the ADHDC group. The subjects with missing symptoms severity score were included 29 from ADHDI group, 11 from ADHDC group and 36 from TDC group. In contrast, there was no missing age information.

**Table 7:** **Structural data Pearson correlation coefficients of the 12 regions with age under consideration**.

	**TDC**		**ADHDC**		**ADHDI**	
	***p*-value**	**Rho**	***p*-value**	**Rho**	***p*-value**	**Rho**
**LEFT HEMISPHERE**						
Superior frontal sulcus, MFG	0.1372	−0.2069	0.7824	0.0388	0.4319	0.1103
Precentral gyrus	0.1432	−0.2038	0.4745	−0.1001	0.9545	−0.008
Postcentral gyrus (middle)	0.0566	−0.2635	0.6064	0.0724	0.7336	−0.0479
Postcentral gyrus (dorsal)	0.0871	−0.2373	0.8634	−0.0242	0.0158	−0.3298
MTG	0.0001^*^	−0.5326	0.4412	−0.1080	0.2892	−0.1483
Orbital gyri, olfactory sulcus	0.0024	−0.4071	0.0003	−0.4711	0.0277	−0.3024
fusiform gyrus	0.0011	−0.4360	0.0031	−0.3986	0.6097	−0.0717
Temporal pole	0.2006	0.1786	0.5023	−0.0942	0.3713	−0.1253
Intraparietal sulcus	0.1146	−0.2193	0.3500	−0.1309	0.1211	−0.2156
SFG	0.0001^*^	−0.4978	0.3447	−0.1324	0.5520	−0.0836
**RIGHT HEMISPHERE**						
Precentral gyrus/Central sulcus	0.0386	−0.2849	0.6293	0.0678	0.0482	0.2727
SFG	0.2548	0.1592	0.3201	0.1392	0.5198	0.0904

*TDC, typically developing children; ADHDI, attention-deficit/hyperactivity disorder, inattentive type; ADHDC, attention-deficit/hyperactivity disorder, combined type; MFG, middle frontal gyrus; MTG, middle temporal gyrus; SFG, superior frontal gyrus*.

* *Result has a significant correlation coefficient: (corrected at p < 0.000463)*.

### Correlation coefficient: functional data

For the functional data, the beta value for each of the ANOVA-based significant cluster was calculated by using AFNI program 3dmaskave. The Pearson correlation coefficients were calculated between the beta values and age, IQ, and symptoms severity scores.

After applying the Bonferroni correction method with *p* < 0.000617, no significant region was found.

### Significant features

Highest subgroup classification accuracies were obtained by using the significant features as shown in Table S2 (Supplementary Material).

## Discussion

This study reports the multi-modal, multi-measure, and multi-class classification results on the ADHD-200 dataset. We calculated and used the global connectivity maps from the functional part of the dataset as classification features. Atlas-based anatomical measures from the structural part of the dataset were also used as features for classification. A hierarchical feature extraction framework is proposed in this study to extract the features with high prediction score. An ELM classifier was used to classify the data. The ELM classifier in combination with the proposed feature extraction framework outperforms the ELM without feature extraction and two SVMs with the proposed feature extraction framework.

The proposed hierarchical feature extraction method enables us to achieve high and significantly accurate classification accuracies. As a result, we achieved as high as 76.190% (*p* < 0.0001) accuracy in multi-class settings. To the best of our knowledge, this is the highest test accuracy reported on the ADHD-200 dataset. Therefore, the multi-modal, multi-measure and multi-class classification was an effective approach to achieve high discrimination scores.

The ELM classifier in combination with the sparse feature autoencoder works very well for the classification of neuroimaging data such as in a recent previous work (Qureshi et al., 2016) based on hierarchical extreme learning machine (H-ELM) (Tang et al., 2016) classification framework. However, the current study proposed a more simplified sparse feature extraction framework that enables us to acquire higher classification accuracies.

### Comparison with previous work

In recent years, many studies about classification-based diagnosis of ADHD have been reported. Most of these studies were binary and the classification scores were ranging from about 72 to 95% (Igual et al., 2012; Lim et al., 2013; Peng et al., 2013; Johnston et al., 2014; Deshpande et al., 2015; Hammer et al., 2015; Iannaccone et al., 2015; Wolfers et al., 2015; Qureshi and Lee, 2016; Qureshi et al., 2016; Xiao et al., 2016). Except for one study, all used feature selection methods prior to classification (Wolfers et al., 2015). In Xiao et al. (2016), a different ADHD dataset was used with only 47 subjects, compared to the current study. Although the theme of this study by Xiao et al. (2016) is similar to our present work, the results are not comparable due to dataset differences and their use of only cortical thickness ROIs. In addition, our main focus was the multi-class, multi-measure, and multi-modal classification of the ADHD-200 dataset. Previously, a very few multi-class (Fair et al., 2012; Qureshi et al., 2016) and multi-modal (Colby et al., 2012; Dai et al., 2012) classification studies have been published with ADHD-200 dataset. However, to the best of our knowledge, the present study is the first multi-modal, multi-measure, and multi-class classification study of the ADHD-200 dataset. In addition, the multi-class classification accuracy of 76.190% (*p* < 0.0001) is the highest reported accuracy on the ADHD-200 dataset in multi-class settings. However, all the aforementioned studies, except a recent study (Qureshi et al., 2016), used different subject selection compared to us. Therefore, the present results are not comparable to the previous studies. The multi-class classification score of the structural part 73.810% (*p* < 0.0001) of the present study is much higher than the results (60.78%) in Qureshi et al. (2016), which utilized the exact same subject selection for classification. The present study is also comparable to Qureshi et al. (2016) in that it also utilized sparse hierarchical feature extraction framework with basic ELM, while Qureshi et al. (2016) used H-ELM classification framework, which is a similar approach in principal. However, the method of the present study outperformed all the results in Qureshi et al. (2016) because the proposed hierarchical feature extraction framework can extract features with the higher predictive power as compared to the sparse autoencoder. In addition, in the present study we utilized many additional feature measures that were not used in Qureshi et al. (2016). We believe that these measures played a vital role in increasing the classification accuracy. However, the exact causes of the boosted accuracy are not yet determined.

### Correlation analysis of the significant regions

We two significant negative Pearson correlation coefficients between the cortical thickness of the regions and age. During adolescence, normal children undergo the cortical thinning phase (Sowell et al., 2004). Consistent with these findings, the two cortical regions in our results showed a decreasing tendency of cortical thickness with age in the TDC group. However, this pattern seemed to be weaker in the ADHDC and ADHDI groups: no regions in each had negative correlation coefficients. In other words, the patients exhibited some deficits in cortical thinning in the middle temporal gyrus and superior frontal gyrus. While considering the middle temporal gyrus; it can be included in ventral attention networks and superior frontal gyrus is related to the dorsal attention networks, therefore, the structural thinning deficits might be used as important markers to discriminate patients and controls (Aboitiz et al., 2014). Thus, we postulate that normal cortical thinning development was interrupted in the ADHD groups. Based on previous findings suggesting that ADHD is related to cortical thinning, the patients might experience abrupt thinning of some brain regions related to attention and motor functions (Makris et al., 2007; Narr et al., 2009).

Moreover, it is evident from our classification results that those significant statistical differences may underlie the high classification accuracy. The results in Vaidya (2011), McLeod et al. (2014), Tegelbeckers et al. (2015) further endorse our results of the correlation analysis of ANOVA-based significant regions with age.

### Clinical relevance of statistical results

The significant regions from both structural and functional ANOVA results play an important role in acquiring highly accurate differential diagnosis when used in combination with the ROI based significant features that did not form part of the outcome of the ANOVA analysis. The following description of the regions obtained from our ANOVA analysis may have clinical significance:

Most of the neuroimaging studies addressing ADHD emphasize that the selected regions in the present study has a strong connection with the neurodevelopmental basis of this disorder. Specifically, the cortical thinning problem and the attention and motor network hypoconnectivity have been suggested to be involved in ADHD (McCarthy et al., 2014; McLeod et al., 2014). These regions showed significant differences between the ADHD and control groups (Qureshi et al., 2016). The functional ANOVA results again endorse the significance of our noteworthy results. A few of the significant regions from our functional ANOVA results overlap with those of structural ANOVA results. They include the left precentral gyrus, the right precentral gyrus, the left middle frontal gyrus, and the right superior temporal gyrus, which are all related to the motor or attention networks.

Our significant feature results showed that structural information is more crucial for the classification. It means that combined gross classification by sMRI and supporting information from fMRI can increase accuracy. In our structural ANOVA results, multiple areas of the frontal cortex, including the middle frontal gyrus (MFG), the precentral gyrus, and the postcentral gyrus in the parietal cortex, showed significantly higher cortical thickness in the TDC group. Our findings are in accordance with previous results (Durston, 2003; Shaw et al., 2007; Valera et al., 2007), and this might contribute to the high classification accuracy of our model. On the other hand, although not much is known about the temporal lobe (Lorberboym et al., 2004), it is evident that the cortical thickness differs significantly in ADHD patients as compared to the normal control group (Fernández-Jaén et al., 2014). Cortical thickness changes over time, therefore we focused on the dynamic changes of thickness according to the ages. Generally, ADHD has cortical thinning compared to controls after development. In Narr et al. (2009) the mean age was of 11.7 years. However, ADHD has cortical thinning problems during the development which are related to the delayed cortical maturation. The mean age of peak brain thickness is 10.5 years for ADHD and 7.5 years for controls. It means after 10.5 years of age, ADHD experiences fast decreases of cortical thickness while controls have enough time to maturate the brain. In line with these evidences, we can say that ADHD has delayed cortical thinning, but finally they can get thinner cortex than controls. In addition, many brain areas can maturate with different speeds, therefore that the results were confusing for us. Our results from the functional ANOVA reconfirm this finding. The middle temporal gyrus is associated with language abilities, visual perception, multi-modal sensory integration, and semantic memory processing (Qureshi et al., 2016). Our findings from the functional ANOVA result are consistent with these results. The bilateral postcentral gyrus (Martinussen et al., 2005), right declive (Ortiz et al., 2015), and Left middle occipital gyrus were reported to be affected in ADHD. Visual perception ability is also related to the visuospatial working memory which is the common symptom of ADHD (Valera et al., 2010; Vaidya, 2011; McLeod et al., 2014). ADHD is related to the semantic memory and language problems (Felton et al., 1987). Our functional ANOVA results endorse these findings, thus making our observations highly beneficial to clinicians.

## Conclusions

This study reports the correlation between both structural and functional measures as well as age IQ and symptom severity score. However, IQ and symptoms severity score information, which is also a very important parameter to determine the correlation among different groups, was missing for a few subjects in the original dataset. In addition, the exact cause of the boosted accuracy was unknown. This is a major limitation of this study.

In conclusion, we found that the proposed hierarchical feature extraction model in combination with ELM serves very well both the binary and the multi-class classification of ADHD. In the future, we will perform linear mixed effect modeling and multi-variate modeling on the selected data of the ADHD-200 dataset in order to acquire the significant regions using both neuroimaging (structural and functional) data and age information.

## Author contributions

MQ selected the subjects for balanced experiment design from the publicly available ADHD-200 database, pre-processed the data, developed, and applied the feature selection methods, classified the data, and found the significant clinical biomarkers. JO, BM, HJ, and BL helped in drafting the introduction and discussion parts of the manuscript with MQ. BL supervised the entire research process and revised the manuscript for publication. All authors contributed to the research design, results interpretation, and proofreading of the final manuscript.

## Funding

This research was supported by a grant of the Korea Health Technology R&D Project through the Korea Health Industry Development Institute (KHIDI), funded by the Ministry of Health & Welfare, Republic of Korea (grant number: HI16C0132). This work was also supported by the GIST Research Institute (GRI) in 2017.

### Conflict of interest statement

The authors declare that the research was conducted in the absence of any commercial or financial relationships that could be construed as a potential conflict of interest.
