# Corrigendum: Multi-modal, Multi-measure, and Multi-class Discrimination of ADHD with Hierarchical Feature Extraction and Extreme Learning Machine Using Structural and Functional Brain MRI

**DOI:** 10.3389/fnhum.2017.00292

**Published:** 2017-05-31

**Authors:** Muhammad Naveed Iqbal Qureshi, Jooyoung Oh, Beomjun Min, Hang Joon Jo, Boreom Lee

**Affiliations:** ^1^Department of Biomedical Science and Engineering, Institute of Integrated Technology, Gwangju Institute of Science and TechnologyGwangju, South Korea; ^2^Department of Neuropsychiatry, Seoul National University HospitalSeoul, South Korea; ^3^Department of Neurologic Surgery, Mayo ClinicRochester, MN, United States

**Keywords:** ADHD-200, global functional connectivity, neuroimaging, ANOVA, machine learning, revised recursive feature elimination, hierarchical feature extraction, extreme learning machine

In the original article, there was a mistake in “TABLE 6 | Binary classification results” as published. We made errors while recording the supporting result values of sensitivity, specificity, F1-score, and precision. However, the main results of accuracy remain intact. To ensure the correctness and reproducibility of the results, we calculated all of these measures again. In addition, sensitivity, and recall represent the same measure, therefore, we omit the recall results. The corrected “TABLE 6 | Binary classification results” appears below. The authors apologize for this error and state that this does not change the scientific conclusions of the article in any way.

**Table 6:** **Binary classification results**.

**Classifier**		**Group**		
		**ADHDC-TDC**	**ADHDC-ADHDI**	**ADHDI-TDC**
ELM	Accuracy (%)	89.286	85.714	**92.857**
	*p*-value	<0.0001	<0.0001	<**0.0001**
	Sensitivity	86.667	77.778	**100.00**
	Specificity	92.310	100.00	**87.500**
	F1-Score	89.655	87.500	**92.307**
	Precision	92.857	100.00	**85.714**
ELM-NFS	Accuracy (%)	71.429	67.857	67.857
	*p*-value	<0.0351	<0.0348	<0.0343
	Sensitivity	100.00	77.780	69.231
	Specificity	63.641	63.160	66.667
	F1-Score	60.000	60.870	66.667
	Precision	42.857	50.000	64.290
SVM linear	Accuracy (%)	71.429	82.143	67.857
	Sensitivity	75.000	76.471	61.900
	Specificity	68.750	90.910	85.714
	F1-Score	69.231	83.869	74.290
	Precision	64.286	92.857	92.857
SVM-RBF	Accuracy (%)	53.571	57.143	60.714
	Sensitivity	53.333	55.556	66.667
	Specificity	53.850	60.000	57.894
	F1-Score	55.170	62.500	52.170
	Precision	57.140	71.429	42.860

*ELM, extreme learning machine; TDC, typically developing children; ADHDI, attention deficit/hyperactivity disorder-inattentive type; ADHDC, attention-deficit/hyperactivity disorder combined type; SVM, support vector machine; RBF, radial basis function; NFS, no feature selection applied. Besides the ELM-NFS all the three (ELM, SVM linear, and SVM-RBF) based classification scores were obtained with the most discriminative features selected through the hierarchical feature selection method. Bold values represents the highest accuracy and its corresponding evaluation measures*.

## Conflict of interest statement

The authors declare that the research was conducted in the absence of any commercial or financial relationships that could be construed as a potential conflict of interest.

