# Supplementary material for: Multi-modal, Multi-measure, and Multi-class Discrimination of ADHD with Hierarchical Feature Extraction and Extreme Learning Machine Using Structural and Functional Brain MRI
Source: Front Hum Neurosci. 2017 Apr 4;11:157. doi: 10.3389/fnhum.2017.00157 (PMC5378777; doi:10.3389/fnhum.2017.00157)
Supplement: Supplementary file 1 [file Table1.pdf]

Supplementary Material

Multi-modal, Multi-measure, and Multi-class Discrimination of ADHD with Hierarchical Feature Extraction and Extreme Learning Machine Using Structural and Functional Brain MRI

Muhammad Naveed Iqbal Qureshi [1], Jooyoung Oh [1], Beomjun Min [2], Hang Joon Jo [3], and Boreom Lee[1*]

[1]Department of Biomedical Science and Engineering, Institute of Integrated Technology, Gwangju Institute of Science and Technology, Gwangju, Korea, [2]Department of Neuropsychiatry, Seoul National University Hospital, Seoul, Korea, [3]Department of Neurologic Surgery, Mayo Clinic, Rochester, Minnesota, USA

*** Correspondence:**
Boreom Lee
leebr@gist.ac.kr, Phone: +82-62-715-3272, Fax: +82-62-715-3244

1 Supplementary Tables

Table S-I. Anatomical name and indices of all the atlas-ROI based features

Feature Measure	fMRI		C-Thickness		C-Area		C-Volume		C-Curvature		C-Thick-SD		Sub-C Volume		WM Volume		Intensity		Volume all	
Indices	1-102		103-166		167-230		231-292		293-354		355-416		417-453		454-521		522-561		562-577	
Hemisphere	lh	rh	lh	rh	lh	rh	lh	rh	lh	rh	lh	rh	lh	rh	lh	rh	lh	rh	lh	rh
Anatomical Region																				
Banks superior temporal sulcus	1	35	-	-	-	-	-	-	-	-	-	-	-	-	454	488	-	-	-	-
Caudal anterior cingulate	2	36	103	135	167	199	231	262	293	324	355	386	-	-	455	489	-	-	-	-
Caudal middle frontal gyrus	3	37	104	136	168	200	232	263	294	325	356	387	-	-	456	490	-	-	-	-
Cuneus	4	38	105	137	169	201	233	264	295	326	357	388	-	-	457	491	-	-	-	-
Entorhinal cortex	5	39	106	138	170	202	234	265	296	327	358	389	-	-	458	492	-	-	-	-
Fusiform gyrus	6	40	107	139	171	203	235	266	297	328	359	390	-	-	459	493	-	-	-	-

Feature Measure	fMRI		C-Thickness		C-Area		C-Volume		C-Curvature		C-Thick-SD		Sub-C Volume		WM Volume		Intensity		Volume all	
Indices	1-102		103-166		167-230		231-292		293-354		355-416		417-453		454-521		522-561		562-577	
Hemisphere	lh	rh	lh	rh	lh	rh	lh	rh	lh	rh	lh	rh	lh	rh	lh	rh	lh	rh	lh	rh
Anatomical Region																				
Inferior parietal lobule	7	41	108	140	172	204	236	267	298	329	360	391	-	-	460	494	-	-	-	-
Inferior temporal gyrus	8	42	109	141	173	205	237	268	299	330	361	392	-	-	461	495	-	-	-	-
Isthmus cingulate	9	43	110	142	174	206	238	269	300	331	362	393	-	-	462	496	-	-	-	-
Lateral occipital cortex	10	44	111	143	175	207	239	270	301	332	363	394	-	-	463	497	-	-	-	-
Lateral orbitofrontal gyrus	11	45	112	144	176	208	240	271	302	333	364	395	-	-	464	498	-	-	-	-
Lingual gyrus	12	46	113	145	177	209	241	272	303	334	365	396	-	-	465	499	-	-	-	-
Medial orbitofrontal gyrus	13	47	114	146	178	210	242	273	304	335	366	397	-	-	466	500	-	-	-	-
Middle temporal gyrus	14	48	115	147	179	211	243	274	305	336	367	398	-	-	467	501	-	-	-	-
Parahippocampal gyrus	15	49	116	148	180	212	244	275	306	337	368	399	-	-	468	502	-	-	-	-
Paracentral gyrus	16	50	117	149	181	213	245	276	307	338	369	400	-	-	469	503	-	-	-	-
Pars opercularis (IFG)	17	51	118	150	182	214	246	277	308	339	370	401	-	-	470	504	-	-	-	-
Pars orbitalis (IFG)	18	52	119	151	183	215	247	278	309	340	371	402	-	-	471	505	-	-	-	-
Pars triangularis (IFG)	19	53	120	152	184	216	248	279	310	341	372	403	-	-	472	506	-	-	-	-
Pericalcarine cortex	20	-	121	153	185	217	249	280	311	342	373	404	-	-	473	507	-	-	-	-
Postcentral gyrus	21	54	122	154	186	218	250	281	312	343	374	405	-	-	474	508	-	-	-	-
Posterior cingulate gyrus	22	55	123	155	187	219	251	282	313	344	375	406	-	-	475	509	-	-	-	-
Precentral gyrus	23	56	124	156	188	220	252	283	314	345	376	407	-	-	476	510	-	-	-	-
Precuneus	24	57	125	157	189	221	253	284	315	346	377	408	-	-	477	511	-	-	-	-
Rostral anterior cingulate	25	58	126	158	190	222	254	285	316	347	378	409	-	-	478	512	-	-	-	-
Rostral middle frontal	26	59	127	159	191	223	255	286	317	348	379	410	-	-	479	513	-	-	-	-
Ssuperior frontal	27	60	128	160	192	224	256	287	318	349	380	411	-	-	480	514	-	-	-	-
Superior parietal lobule	28	61	129	161	193	225	257	288	319	350	381	412	-	-	481	515	-	-	-	-
Superior temporal gyrus	29	62	130	162	194	226	258	289	320	351	382	413	-	-	482	516	-	-	-	-
Supramarginal gyrus	30	63	131	163	195	227	259	290	321	352	383	414	-	-	483	517	-	-	-	-
Frontal pole	31	64	-	-	-	-	-	-	-	-	-	-	-	-	484	518	-	-	-	-
Temporal pole	32	65	-	-	-	-	-	-	-	-	-	-	-	-	485	519	-	-	-	-
Transverse temporal gyrus	33	66	132	164	196	228	260	291	322	353	384	415	-	-	486	520	-	-	-	-

Feature Measure	fMRI		C-Thickness		C-Area		C-Volume		C-Curvature		C-Thick-SD		Sub-C Volume		WM Volume		Intensity		Volume all	
Indices	1-102		103-166		167-230		231-292		293-354		355-416		417-453		454-521		522-561		562-577	
Hemisphere	lh	rh	lh	rh	lh	rh	lh	rh	lh	rh	lh	rh	lh	rh	lh	rh	lh	rh	lh	rh
Anatomical Region																				
Insula	34	67	133	165	197	229	261	292	323	354	385	416	-	-	487	521	-	-	-	-
Mean Thickness	-	-	134	166	-	-	-	-	-	-	-	-	-	-	-	-	-	-	-	-
White Surface Area	-	-	-	-	198	230	-	-	-	-	-	-	-	-	-	-	-	-	-	-
Cerebral White Matter	68	84	-	-	-	-	-	-	-	-	-	-	-	-	-	-	-	-	-	-
Lateral Ventricle	69	85	-	-	-	-	-	-	-	-	-	-	417	434	-	-	522	540	-	-
Inferior Lateral Ventricle	-	-	-	-	-	-	-	-	-	-	-	-	418	435	-	-	523	541	-	-
Cerebellum White Matter	70	86	-	-	-	-	-	-	-	-	-	-	419	436	-	-	524	542	-	-
Cerebellum Cortex	71	87	-	-	-	-	-	-	-	-	-	-	420	437	-	-	525	543	-	-
Thalamus Proper	72	88	-	-	-	-	-	-	-	-	-	-	421	438	-	-	526	544	-	-
Caudate	73	89	-	-	-	-	-	-	-	-	-	-	422	439	-	-	527	545	-	-
Putamen	74	90	-	-	-	-	-	-	-	-	-	-	423	440	-	-	528	546	-	-
Pallidum	75	91	-	-	-	-	-	-	-	-	-	-	424	441	-	-	529	547	-	-
3rd-Ventricle	76		-	-	-	-	-	-	-	-	-	-	425		-	-	530		-	-
4th-Ventricle	77		-	-	-	-	-	-	-	-	-	-	426		-	-	531		-	-
Brain-Stem	78		-	-	-	-	-	-	-	-	-	-	427		-	-	532		-	-
Hippocampus	79	92	-	-	-	-	-	-	-	-	-	-	428	442	-	-	533	548	-	-
Amygdala	80	93	-	-	-	-	-	-	-	-	-	-	429	443	-	-	534	549	-	-
CSF	-	-	-	-	-	-	-	-	-	-	-	-	430		-	-	535		-	-
Accumbens area	81	94	-	-	-	-	-	-	-	-	-	-	431	444	-	-	536	-	-	-
Ventral diencephalon	82	95	-	-	-	-	-	-	-	-	-	-	432	445	-	-	537	-	-	-
Vessel	-	-	-	-	-	-	-	-	-	-	-	-	-	-	-	-	538	-	-	-
Choroid-plexus	83	96	-	-	-	-	-	-	-	-	-	-	433	446	-	-	539	-	-	-
WM hypointensities	-	-	-	-	-	-	-	-	-	-	-	-	447		-	-	554		-	-
Non WM hypointensities	-	-	-	-	-	-	-	-	-	-	-	-	-	-	-	-	555		-	-
Optic Chiasm	97		-	-	-	-	-	-	-	-	-	-	448		-	-	556		-	-
Posterior CC	98		-	-	-	-	-	-	-	-	-	-	449		-	-	557		-	-

Feature Measure	fMRI		C-Thickness		C-Area		C-Volume		C-Curvature		C-Thick-SD		Sub-C Volume		WM Volume		Intensity		Volume all	
Indices	1-102		103-166		167-230		231-292		293-354		355-416		417-453		454-521		522-561		562-577	
Hemisphere	lh	rh	lh	rh	lh	rh	lh	rh	lh	rh	lh	rh	lh	rh	lh	rh	lh	rh	lh	rh
Anatomical Region																				
Middle Posterior CC	99		-	-	-	-	-	-	-	-	-	-	450		-	-	558		-	-
Central CC	100		-	-	-	-	-	-	-	-	-	-	451		-	-	559		-	-
Middle Anterior CC	101		-	-	-	-	-	-	-	-	-	-	452		-	-	560		-	-
Anterior CC	102		-	-	-	-	-	-	-	-	-	-	453		-	-	561		-	-
Brain Segment Volume	-	-	-	-	-	-	-	-	-	-	-	-	-	-	-	-	-	-	562	
Brain Segment Vol Not Ventricle	-	-	-	-	-	-	-	-	-	-	-	-	-	-	-	-	-	-	563	
Brain Segment Vol Not Vent Surf	-	-	-	-	-	-	-	-	-	-	-	-	-	-	-	-	-	-	564	
Cortex Vol	-	-	-	-	-	-	-	-	-	-	-	-	-	-	-	-	-	-	565	566
Whole Cortex Vol	-	-	-	-	-	-	-	-	-	-	-	-	-	-	-	-	-	-	567	
Cortical White Matter Vol	-	-	-	-	-	-	-	-	-	-	-	-	-	-	-	-	-	-	568	569
Whole Cortical White Matter Vol	-	-	-	-	-	-	-	-	-	-	-	-	-	-	-	-	-	-	570	
Sub-Cortical Gray Vol	-	-	-	-	-	-	-	-	-	-	-	-	-	-	-	-	-	-	571	
Total Gray Vol	-	-	-	-	-	-	-	-	-	-	-	-	-	-	-	-	-	-	572	
SupraTentorial Vol	-	-	-	-	-	-	-	-	-	-	-	-	-	-	-	-	-	-	573	
SupraTentorial Vol Not Vent	-	-	-	-	-	-	-	-	-	-	-	-	-	-	-	-	-	-	574	
SupraTentorial Vol Not Vent Vox	-	-	-	-	-	-	-	-	-	-	-	-	-	-	-	-	-	-	575	
Brain Mask Volume	-	-	-	-	-	-	-	-	-	-	-	-	-	-	-	-	-	-	576	
Estimated Total ICV	-	-	-	-	-	-	-	-	-	-	-	-	-	-	-	-	-	-	577	

Abbreviation: IFG = Inferior frontal gyrus; WM = white matter; CC = corpus callosum; ICV = intra cranial volume; CSF = cerebro-spinal fluid; WM = white matter; C = cortical; SD = standard deviation. Complete feature vector of all the multimodal and multi-measure features includes the ANOVA-ROI based features in addition to the atlas-ROI based features. ANOVA-ROI based features were concatenated after the 577 atlas-ROI based features, such that:

598 (all feature vector) = 577 (atlas-ROI based) + 9 (fMRI-ANOVA ROI) + 12 (sMRI-ANOVA ROI)

frontiers

Table S-II. Significant features for acquiring the highest discrimination from each of the binary group

Subgroup		ADHDC-ADHDI		ADHDI-TDC		ADHDC-TDC	
No	Name	Measure	Frequency	Measure	Frequency	Measure	Frequency
1	Caudal anterior cingulate	T, TS	3	T	2	T	1
2	Caudal middle frontal gyrus	T	2	T	1	-	-
3	Entorhinal cortex	T, TS	4	T,TS	3	-	-
4	Fusiform gyrus	T	2	T	2	-	-
5	Inferior parietal lobule	T	2	T	1	-	-
6	Inferior temporal gyrus	T, TS	4	T	2	-	-
7	Isthmus cingulate	T, TS	4	T,TS	3	-	-
8	Lateral orbitofrontal gyrus	T, TS	4	T	2	-	-
9	Medial orbitofrontal gyrus	T, TS	4	T	2	-	-
10	Middle temporal gyrus	T	2	T	2	T	2
11	Parahippocampal gyrus	T	2	T	2	-	-
12	Paracentral gyrus	T	2	T	1	-	-
13	Pars opercularis (IFG)	T	2	T	2	-	-
14	Pars orbitalis (IFG)	T	2	T	2	-	-
15	Pars triangularis (IFG)	T	2	T	2	-	-
16	Posterior cingulate	T, TS	4	T	2	-	-
17	Precentral gyrus	T	2	-	-	-	-
18	Precuneus gyrus	T	2	-	-	-	-
19	Rostral anterior cingulate	T	2	T	2	T	2
20	Rostral middle frontal	T	2	-	-	-	-
21	Superior frontal	T,V,WM	6	T,V,WM	6	V,WM	4
22	Superior parietal lobule	T	1	-	-	-	-
23	Superior temporal gyrus	T	2	T	2	T	2
24	Supramarginal gyrus	T	2	T	2	-	-
25	Transverse temporal gyrus	T	2	-	-	-	-
26	Insula	T, TS	4	T	2	T	2

Subgroup		ADHDC-ADHDI		ADHDI-TDC		ADHDC-TDC	
No	Name	Measure	Frequency	Measure	Frequency	Measure	Frequency
27	Mean thickness of cortex	T	2	-	-	-	-
28	White surf area	A	2	A	2	A	2
29	Cerebellum cortex	SV	2	SV	2	SV	2
30	Cerebellum white matter	I	2	I	2	I	2
31	Thalamus proper	I	2	I	2	I	2
32	Caudate	I	2	I	2	-	-
33	Putamen	I	2	I	2	-	-
34	Pallidum	I	2	I	2	I	2
35	Brain-stem	I	1	I	1	I	1
36	Hippocampus	I	2	-	-	-	-
37	Amygdala	I	1	-	-	-	-
38	Accumbens area	I	2	-	-	-	-
39	Ventral diencephalon	I	2	I	2	I	2
40	WM hypointensities	I	1	I	1	-	-
41	Non WM hypointensities	I	1	-	-	-	-
42	Optic chiasm	I	1	I	1	-	-
43	Posterior CC	I	1	I	1	I	1
44	Middle posterior CC	I	1	I	1	I	1
45	Central CC	I	1	I	1	I	1
46	Middle anterior CC	I	1	I	1	I	1
47	Anterior CC	I	1	I	1	I	1
48	Brain segment volume	VA	1	-	-	-	-
49	Brain segment vol not vent	VA	1	-	-	-	-
50	Brain seg vol not vent surf	VA	1	-	-	-	-
51	Supratentorial volume	VA	1	-	-	-	-
52	Supratentorial vol not vent	VA	1	-	-	-	-
53	Supratentorial volume voxel count	VA	1	-	-	-	-
54	Brain Mask volume	VA	1	VA	1	VA	1
55	Estimated total ICV	VA	1	VA	1	VA	1

Abbreviation: IFG = Inferior frontal gyrus; WM = white matter; CC = corpus callosum; ICV = intra cranial volume; T = mean cortical thickness; TS = cortical thickness standard deviation; V = cortical volume; A = cortical surface area; SV = sub-cortical volume; I = intensity; VA = overall volume
